# When loneliness amplifies the risk of problematic emotional dependence: perceived anthropomorphism and basic psychological need satisfaction in AI companion use

**DOI:** 10.3389/fpsyg.2026.1900817

**Published:** 2026-10-13

**Authors:** Ting Liu, Ruey-ming Shih, Tingyun Luo, Shining Jin

**Affiliations:** 1 School of Design, Fujian University of Technology, Fuzhou, China; 2 The Graduate Institute of Design Science, Tatung University, Taipei, Taiwan; 3 Fujian Polytechnic of Information Technology, Fuzhou, China

**Keywords:** AI companion, basic psychological need satisfaction, loneliness, perceived anthropomorphism, problematic emotional dependency, self-determination theory

## Abstract

**Background:**

Companion-oriented conversational AI can be experienced as socially responsive, yet evidence about problematic reliance requires construct clarity and noncausal interpretation. This study examined whether perceived anthropomorphism was associated with problematic AI-companion dependence directly and indirectly through basic psychological need satisfaction.

**Methods:**

A cross-sectional online survey was completed by 666 adults in China who reported using a listed AI companion or conversational AI product during the preceding month. The nonclinical outcome was operationalized with six indicators spanning impaired control, distress, continued use, perceived understanding, emotion-regulation reliance, and displacement of offline communication. Measurement evidence was examined using reliability estimates, one-factor confirmatory models, and HTMT. Standardized composite-score regressions used HC3 robust standard errors, and the indirect association was evaluated with 5,000 bootstrap samples.

**Results:**

Perceived anthropomorphism was positively associated with need satisfaction (*β* = 0.569, *p* < 0.001). In the outcome model, need satisfaction (*β* = 0.317, *p* < 0.001) and perceived anthropomorphism (*β* = 0.385, *p* < 0.001) were both positively associated with problematic dependence. The standardized indirect association was 0.180 (95% bootstrap CI [0.137, 0.229]); *R*^2^ was 0.323 for need satisfaction and 0.389 for problematic dependence.

**Conclusion:**

The pattern is consistent with a need-satisfaction account of companion-oriented AI use, but the cross-sectional design does not establish temporal ordering, substitution, or causality. Design and policy implications should therefore focus on transparency, user agency, and safeguards against escalating reliance rather than treating anthropomorphism itself as inherently harmful.

## 1 Introduction

Conversational artificial intelligence covers a broad set of systems, including general-purpose generative-AI assistants, task-oriented chatbots, and systems designed for sustained relational interaction. In this article, AI companion systems refers to conversational systems designed primarily for continuing, emotionally responsive interaction; companion-oriented conversational AI use refers to a user’s relational use of either a dedicated companion system or a broader conversational AI system; and generative AI/chatbots denotes the wider technological category. This distinction is important because system type, intended function, and actual use context are not interchangeable dimensions of technology acceptance or system quality (Salam et al., 2026; Salam and Farooq, 2026).

AI companion systems can provide continuity, personalized memory, emotional responsiveness, and a sense of social presence. Users have described such systems as attentive and nonjudgmental sources of companionship, support, and self-disclosure (Skjuve et al., 2021; Ta et al., 2020; Xie and Pentina, 2022). These experiences can be beneficial, neutral, or problematic depending on the user and context. Human-like interaction should therefore not be presumed harmful; the empirical question is whether particular perceptions and experiences covary with impaired control, distress, or reliance that displaces valued offline activity.

Perceived anthropomorphism may be relevant because it describes the user’s attribution of human-like appearance, expression, understanding, and emotional responsiveness to an artificial agent (Epley et al., 2007; Waytz et al., 2010). Self-Determination Theory (SDT) offers a complementary account: interaction may feel satisfying when it supports autonomy, relatedness, and competence (Deci and Ryan, 2008; Ryan and Deci, 2000). In a companion-oriented context, anthropomorphic perception and need satisfaction are conceptually related but distinct. The former concerns qualities attributed to the AI, whereas the latter concerns the user’s experienced agency, connection, and efficacy during interaction.

The outcome is termed problematic emotional dependence (PED), not pathological emotional dependency. PED is used here as a nonclinical, continuous self-report construct comprising difficulty reducing use, distress when access is unavailable, continued use despite perceived harm, increasing reliance for emotional stability, and prioritization of AI interaction over communication with people (Goh et al., 2025; Huang and Huang, 2025). The term does not imply a psychiatric diagnosis, prevalence estimate, or validated clinical threshold. The construct is interpreted as a broad, nonclinical measure rather than a diagnostic category.

China provides an informative setting because the sample includes users of both domestic platforms (Maoxiang, Zhumengdao, and Xingye) and international platforms (Replika and Character.AI) within a shared linguistic and cultural context. At the same time, the sample is nonprobabilistic and cannot be treated as nationally representative. Cultural expectations concerning self-disclosure, relational obligation, and technology use may influence how items are interpreted; generalization beyond the surveyed users, China, or the included platforms therefore requires replication (Du and Alm, 2024; Epley et al., 2007; Pan et al., n.d.; Ryan and Deci, 2000).

The revised study asks whether perceived anthropomorphism is associated with PAD directly and whether basic psychological need satisfaction carries a statistical indirect association between them (Folk et al. 2025; Huang and Huang, 2025). In response to concerns about construct validity and causal language, the analyses use a revised five-item outcome, compare global and multidimensional representations of need satisfaction, and describe indirect effects as cross-sectional decompositions rather than evidence of a psychological mechanism.

## 2 Literature review and research hypotheses

### 2.1 AI companions as emotionally immersive social technologies

People often respond socially to computers even while recognizing that the system is not human (Nass and Moon, 2000). In companion systems, social cues are central to the product experience: conversation, memory, affective response, and availability can produce perceived reciprocity and relationship-like continuity (Brandtzaeg et al., 2022; Skjuve et al., 2022; Chaturvedi et al., 2024). However, relational use can also occur on a broader assistant platform. The present unit of analysis is therefore companion-oriented use, not an assumption that every included product is a dedicated AI companion.

Prior research reports emotional support and safe self-disclosure alongside concerns about over-reliance, withdrawal-like distress, and reduced offline functioning (Laestadius et al., 2024; Liu et al., 2024; Zhang et al., 2025). These observations motivate examination of problematic reliance but do not justify treating intensive use or anthropomorphic design as intrinsically disordered. A construct-valid analysis must distinguish perceived human-likeness, satisfying interaction, and dysregulated reliance.

### 2.2 Perceived anthropomorphism and basic psychological need satisfaction

Perceived anthropomorphism concerns the extent to which users experience an AI’s appearance, language, understanding, and emotional responses as human-like (Bartneck et al., 2009; Kühne and Peter, 2023). Such perceptions may make interaction feel more responsive and socially meaningful. Within SDT, autonomy refers to volition and authentic self-expression, relatedness to connection and acceptance, and competence to effective action (Chen et al., 2015; Deci and Ryan, 2008). A user who attributes greater human-likeness to an AI may therefore report greater satisfaction of these needs during interaction. Because the design is cross-sectional, the hypothesis concerns covariation rather than direction of causation.

Self-Determination Theory (SDT) provides a useful framework for explaining why anthropomorphism may foster psychological need satisfaction. SDT posits that human motivation and sustained engagement are shaped by the fulfillment of three basic psychological needs: autonomy, competence, and relatedness (Ryan and Deci, 2000). In AI-mediated interactions, human-like appearance and emotionally responsive feedback may support relatedness by simulating the experience of being recognized, understood, and cared for. Natural language interaction may support autonomy by allowing users to initiate, personalize, and redirect conversations according to their own preferences and goals. Responsive and adaptive feedback may further support competence by making interactions feel effective, fluent, and socially meaningful. Prior research in digital media environments has shown that psychological need satisfaction enhances user engagement, intrinsic motivation, and continued participation (Peng et al., 2012; Przybylski et al., 2010). More recent studies on generative AI and AI companions further suggest that anthropomorphic interaction, emotional responsiveness, and friend-like communication are associated with stronger emotional attachment and dependency-related outcomes (Hu et al., 2023; Robayo-Pinzon et al., 2025). Accordingly, the present study proposes that perceived anthropomorphism in AI companions positively influences users’ psychological need satisfaction, which in turn contributes to continued engagement and dependency-related outcomes.

H1: Perceived anthropomorphism is positively associated with basic psychological need satisfaction among AI companion users.

### 2.3 Need satisfaction and problematic emotional dependence

Need satisfaction is generally adaptive when it emerges from environments that support autonomy, competence, and relatedness in ways that broaden human functioning. However, the same motivational mechanism may become problematic when a digital system becomes the dominant, easiest, or most predictable source of need fulfillment. In AI companion use, need satisfaction may reinforce repeated interaction because users receive immediate relatedness, low-risk self-disclosure, and predictable affirmation. Over time, this may reduce motivation to engage in more effortful interpersonal interactions and foster reliance on AI-mediated need fulfillment (Liu et al., 2025; Peng et al., 2012; Przybylski et al., 2010). The present outcome is therefore renamed problematic emotional dependence rather than pathological emotional dependency. It is a non-clinical self-report composite and must not be interpreted as a diagnosis, disorder, clinical threshold, or prevalence estimate.

This study problematic emotional dependence to describe a dependency pattern that combines behavioral and relational elements. The behavioral element resembles problematic technology use, especially loss of control, withdrawal-like distress, relapse, conflict, and continued use despite negative consequences (Andreassen et al., 2012; Fournier et al., 2023; Kuss et al., 2014). The relational element resembles parasocial attachment, in which individuals experience one-sided intimacy, emotional involvement, and perceived relationship importance with a media figure or artificial partner (Cohen, 2004; Gleason et al., 2017; Rubin and McHugh, 1987). This conceptualization is consistent with emerging evidence that AI companion use can involve emotional attachment, over-reliance, self-disclosure, withdrawal, and risks to offline social functioning, while also recognizing that AI companion dependency is distinctive because the object of dependence is experienced as a responsive social other rather than a neutral tool (Boine, 2023; Hu et al., 2023; Liu et al., 2025; Zhang et al., 2025).

If AI companion interaction satisfies users’ basic psychological needs, users may be more likely to develop problematic emotional dependence because the system becomes a motivational resource that is difficult to relinquish. This argument is not simply about heavy use; rather, it concerns the psychological meaning of use. From a self-determination perspective, digital experiences that satisfy autonomy, competence, and relatedness can support motivation and self-regulation, making them especially reinforcing when they meet needs that are central to social functioning (Johnson et al., 2021; Parent, 2023). In AI companion contexts, this reinforcement may be stronger because the system can provide low-risk disclosure, emotional validation, and a sense of being understood, which may encourage attachment, overreliance, and dependency-like patterns (Fang et al., 2025; Skjuve et al., 2022; Zhang et al., 2025). The second hypothesis therefore proposes that psychological need satisfaction positively predicts problematic emotional dependence.

H2: Basic psychological need satisfaction is positively associated with problematic emotional dependence among AI companion users.

### 2.4 The mediating role of basic psychological need satisfaction

The first two hypotheses imply a mediation mechanism. Perceived anthropomorphism should increase problematic emotional dependence because it makes the AI companion more effective at satisfying users’ basic psychological needs. This pathway is conceptually distinct from a simple direct effect of anthropomorphism on attachment. It does not assume that human-like cues automatically produce dependency. Instead, anthropomorphic perception becomes consequential when it makes the AI interaction feel need-satisfying, personally responsive, and relationally meaningful (Guingrich and Graziano, 2025; Heng and Zhang, 2025; Yu and Lan, 2025).

This mediation argument also explains why some users may interact frequently with AI companions without becoming problematic dependence. If anthropomorphic cues are experienced mainly as entertaining, novel, or aesthetically pleasing, but do not become a primary source of autonomy, competence, or relatedness satisfaction, the motivational reinforcement should be weaker. Prior digital media research similarly suggests that the psychological meaning and motivational quality of use matter more than use intensity alone (Sheldon and Titova, 2023; Throuvala et al., 2019). Dependency risk is therefore expected to emerge when anthropomorphism and need satisfaction align, producing a design–need fit that makes the AI companion psychologically substitutive. The third hypothesis formalizes this indirect pathway.

H3: Basic psychological need satisfaction mediates the positive relationship between perceived anthropomorphism and problematic emotional dependence.

### 2.5 Loneliness as a boundary condition

Loneliness is not merely the absence of social contact. Rather, it refers to a perceived gap between desired and actual social connection, and it is therefore closely related to unmet belongingness and relatedness needs (Baumeister and Leary, 1995; Cacioppo and Hawkley, 2009). This distinction is important for AI companion research because lonely users may not approach AI companions simply for novelty or entertainment. Instead, they may approach them as emotionally safe, nonjudgmental, and continuously available substitutes for human connection (Rubin et al., 1985; Liu et al., 2025; Merrill et al., 2022).

Prior studies provide reasons to expect a moderating role of loneliness. Research on companion chatbots suggests that user outcomes are heterogeneous: some users may experience enhanced social confidence, whereas others may risk further isolation, especially when problematic use and smaller social networks are involved (Liu et al., 2025). A large-scale study of Japanese adults found that the positive association between AI companion use and well-being was strongest among individuals reporting high loneliness, suggesting that lonely users may be especially sensitive to AI-provided emotional support (Nakagomi et al., 2026). However, other evidence also points to potential risks: intensive companion-oriented chatbot use, high self-disclosure, and weak human social support have been associated with lower well-being, and higher chatbot usage has been linked to greater loneliness, emotional dependence, and problematic use over time (Fang et al., 2025; Zhang et al., 2025). These findings suggest a dynamic vulnerability process in which loneliness may make AI-provided need satisfaction more reinforcing, while repeated reliance on AI may further deepen emotional dependence.

For users with low loneliness, need satisfaction from an AI companion may remain supplementary because offline relationships continue to provide meaningful relatedness. For users with high loneliness, however, the same AI-provided need satisfaction may acquire substitutive value because the companion can offer a readily available sense of being heard, accepted, and emotionally recognized (De Freitas et al., 2024; Merrill et al., 2022). This logic is consistent with research showing that AI companions may be especially attractive to lonely users, but also with concerns that digital companionship may reproduce or intensify loneliness when it replaces rather than complements human connection (Jacobs, 2024; Liu et al., 2025). Therefore, the fourth hypothesis predicts that loneliness strengthens the positive relationship between need satisfaction and problematic emotional dependence.

H4: Loneliness moderates the positive relationship between basic psychological need satisfaction and problematic emotional dependence, such that the relationship is stronger among users with higher levels of loneliness.

Integrating the mediation and moderation arguments yields the full conditional process model. If perceived anthropomorphism increases psychological need satisfaction, and loneliness strengthens the effect of need satisfaction on problematic emotional dependence, then the indirect effect of perceived anthropomorphism on problematic emotional dependence should be stronger among lonelier users. This final hypothesis therefore tests whether loneliness changes the strength of the entire design-to-dependency mechanism rather than only a single direct path (Hayes, 2015, 2018). Substantively, this expectation is consistent with evidence that anthropomorphic AI can increase dependency-related outcomes through user satisfaction or emotional attachment, and that loneliness shapes how users respond to conversational AI and AI companions (Heng and Zhang, 2025; Nakagomi et al., 2026; Peng et al., 2025; Yu and Lan, 2025). Accordingly, the fifth hypothesis proposes a moderated mediation effect: the indirect relationship between perceived anthropomorphism and problematic emotional dependence through need satisfaction is stronger when loneliness is higher.

H5: The indirect effect of perceived anthropomorphism on problematic emotional dependence through basic psychological need satisfaction is moderated by loneliness, such that the indirect effect is stronger among users with higher levels of loneliness.

To sum up, the research hypotheses are as displayed in Figure 1.

**Figure 1:**
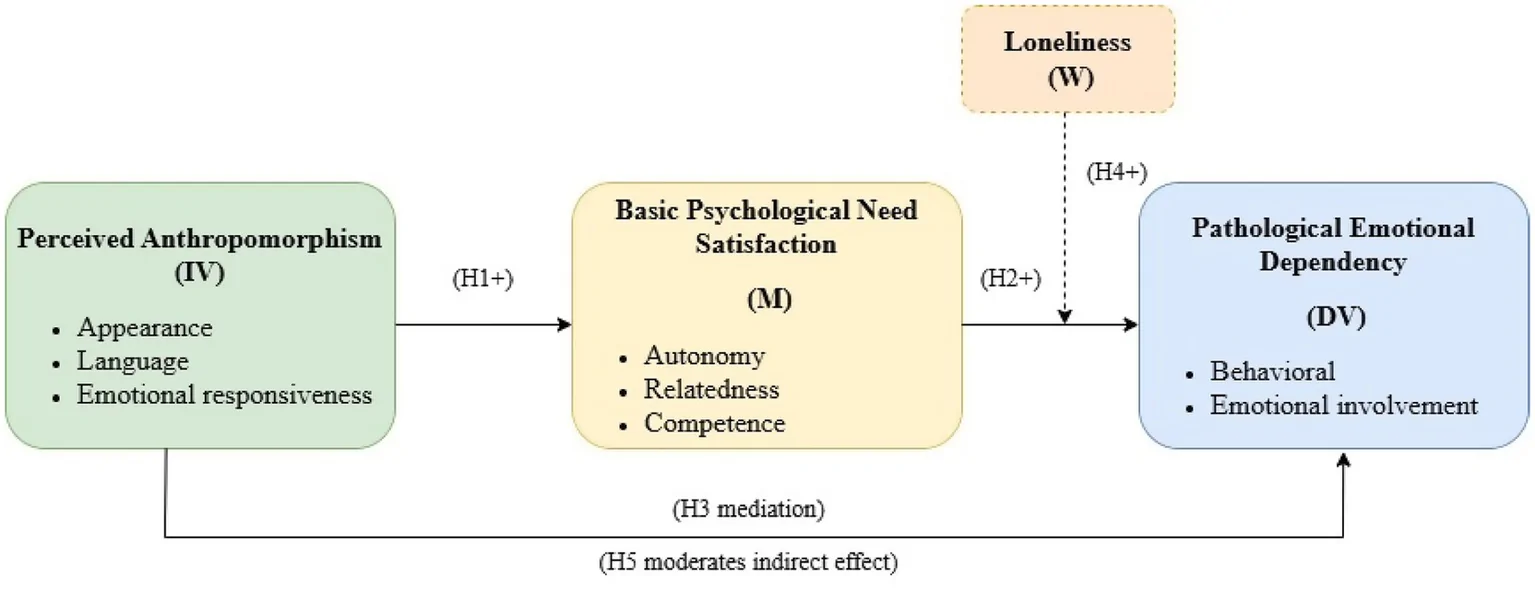
Hypothetical diagram of the study.

## 3 Method

### 3.1 Research design and participants

A three-month online survey was conducted through Wenjuanxing from February to April 2026. Participants were recruited via AI companion user communities on Weibo and Xiaohongshu. Eligible respondents were required to be at least 18 years old, have experience using AI companion applications, and provide informed consent. The questionnaire explained the study purpose, anonymity, voluntary participation, and academic use of the data.

A total of 802 questionnaires were collected. Responses were excluded based on predetermined criteria, including short completion time, failed attention checksor incomplete answers. After excluding 136 invalid responses, 666 valid questionnaires remained, yielding an effective response rate of 83.0%.

A total of 666 valid questionnaires were retained for analysis. As seen from Table 1, the sample included 210 men, 283 women, 123 respondents who selected other gender, and 50 respondents who preferred not to disclose their gender. Respondents were concentrated in younger adult groups, with 283 aged 18 to 25 and 258 aged 26 to 35. The sample nevertheless included older respondents, with 74 aged 36 to 45 and 51 aged 46 or above. The most frequently used AI companion products were Maoxiang, Zhumengdao, Xingye, Replika, Character. AI, and other platforms. The distribution of purposes of use showed that the sample was strongly oriented toward emotional and relational interaction, with role interaction or virtual romantic partnership and psychological support or self-disclosure accounting for the majority of responses.

**Table 1:** Descriptive statistics of respondents and AI companion use.

Variable	Category	Frequency	Percentage
Gender	Male	210	31.5%
	Female	283	42.5%
	Other	123	18.5%
	Prefer not to disclose	50	7.5%
Age	18–25 years	283	42.5%
	26–35 years	258	38.7%
	36–45 years	74	11.1%
	46 years and above	51	7.7%
Weekly usage days	Less than 1 day	60	9.0%
	1–2 days	207	31.1%
	3–4 days	221	33.2%
	5–6 days	93	14.0%
	Every day	85	12.8%
Usage duration	Less than 1 month	46	6.9%
	1–3 months	152	22.8%
	4–6 months	226	33.9%
	7–12 months	147	22.1%
	More than 1 year	95	14.3%
Weekly face-to-face interaction with family/friends	0 days	77	11.6%
	1–2 days	113	17.0%
	3–4 days	177	26.6%
	5–6 days	158	23.7%
	Every day or almost every day	141	21.2%
Most frequently used AI companion product	Maoxiang	114	17.1%
	Zhumengdao	134	20.1%
	Xingye	144	21.6%
	Replika	96	14.4%
	Character.AI	95	14.3%
	Other	83	12.5%
Purpose of use	Emotional companionship	114	17.1%
	Casual chatting/relief from boredom	69	10.4%
	Role interaction/virtual romantic partner	224	33.6%
	Psychological support or self-disclosure	224	33.6%
	Study, work, or information search	24	3.6%
	Other	11	1.7%

### 3.2 Measures

All measures were administered in Chinese using a seven-point Likert response format (1 = strongly disagree, 2 = disagree, 3 = somewhat disagree, 4 = uncertain, 5 = somewhat agree, 6 = agree, 7 = strongly agree). Higher composite scores represented higher levels of the focal construct. The six loneliness items used the same response anchors; LO4-LO6 were positively worded social-connection items and were reverse-scored so that higher values indicated greater loneliness. The delivered analysis file stores these three variables as already direction-aligned scored values, and the stored LOM variable equals the mean of LO1-LO6; they were therefore not reversed a second time.

Perceived anthropomorphism (PA) was measured with six adapted items concerning human-like appearance, language, understanding, and emotional responsiveness (Bartneck et al., 2009; Waytz et al., 2010). It represents perceived humanness of the AI interaction, not an objective design manipulation.

Basic psychological need satisfaction (NS) was measured with six adapted items from the Basic Psychological Need Satisfaction and Frustration Scale, with two items each for autonomy, relatedness, and competence satisfaction in the AI interaction (Chen et al., 2015). The reported model used a total composite. Because relatedness is theoretically central and each dimension has only two indicators, we compared broad and correlated-dimension specifications and estimated the three dimension scores jointly before making dimension-general claims.

Problematic emotional dependence (PED) was measured with the six items previously labeled pathological emotional dependency. The revised label is deliberately non-clinical. PD1-PD3 concern behavioral dysregulation; PD4-PD6 concern relational salience and emotionally consequential reliance. No clinical threshold, diagnosis, or criterion validity is claimed. The item labels are retained as PD1-PD6 to preserve correspondence with the original dataset.

Loneliness was measured with six adapted UCLA Loneliness Scale items (Russell, 1996; Hughes et al., 2004). Attachment anxiety was measured with six adapted ECR/ECR-R anxiety items and was used only as an exploratory alternative moderator (Brennan et al., 1998; Fraley et al., 2000). Attachment anxiety was selected because relational insecurity is conceptually adjacent to, but distinct from, perceived social disconnection.

A covariate-adjusted sensitivity model included gender, age, weekly AI-use frequency, duration of use, weekly face-to-face interaction with family or friends, most-used platform, and recorded primary purpose. Focal adjusted coefficients and model fit are reported in Section 4.3; the complete dummy-coded coefficient matrix is retained with the analysis output, as seen from Tables 2, 3.

**Table 2:** Measurement items, dimensions, and source rationale.

Construct	English item	Scale source
Perceived Anthropomorphism (PA)	PA1. My AI companion looks like a real person.	Adapted from the Godspeed Anthropomorphism Scale and anthropomorphism measures (Bartneck et al., 2009; Waytz et al., 2010).
	PA2. The appearance of my AI companion makes me feel that it has human-like characteristics.	
	PA3. My AI companion expresses itself in a natural, human-like way.	
	PA4. My AI companion can understand the subtle meanings in what I say.	
	PA5. My AI companion can understand and respond appropriately to my emotions.	
	PA6. The emotional expressions of my AI companion make me feel that it truly cares about me.	
Basic psychological need Satisfaction (NS)	NS1. When interacting with my AI companion, I feel free to express myself in my own way.	Adapted from the Basic Psychological Need Satisfaction and Frustration Scale, autonomy satisfaction subscale (Chen et al., 2015).
	NS2. I feel that my interactions with my AI companion are consistent with my true thoughts and feelings.	
	NS3. When interacting with my AI companion, I feel a genuine sense of connection.	
	NS4. When interacting with my AI companion, I feel accepted and accompanied.	
	NS5. When interacting with my AI companion, I feel capable of expressing myself effectively.	
	NS6. I feel that I can interact with my AI companion with ease and competence.	
Problematic emotional dependence (PED)	PED1. I have tried to reduce my use of my AI companion but have not succeeded.	Adapted from behavioral-addiction/problematic-use scales capturing relapse or failed control attempts (Andreassen et al., 2012; Yu et al., 2024; Maral et al., 2025).
	PED2. When I cannot use my AI companion, I feel irritable or anxious.	
	PED3. Even when I know that excessive use of my AI companion is not good for me, I still find it difficult to control my use.	
	PED4. I feel that my AI companion really understands me as a person.	
	PED5. I have developed a special emotional attachment to my AI companion and increasingly need it to maintain my emotional stability.	
	PED6. Sometimes I prioritize interacting with my AI companion over communicating with real people.	
Loneliness (LO)	LO1. How often do you feel that you lack companionship?	Adapted from the UCLA Loneliness Scale Version 3 and the Three-Item Loneliness Scale (Russell, 1996; Hughes et al., 2004).
	LO2. How often do you feel isolated from others?	
	LO3. How often do you feel distant from others?	
	LO4. How often do you feel that there is someone you can talk to?	
	LO4. How often do you feel that you are part of a group of friends?	
	LO5. How often do you feel that you are part of a group of friends?	
	LO6. How often do you feel that you have things in common with the people around you?	
Attachment anxiety (AA)	AA1. I worry about being abandoned by people I care about.	Adapted from attachment-anxiety measures, especially the ECR/ECR-RS anxiety items (Brennan et al., 1998; Fraley et al., 2000)
	AA2. I worry that close others do not care about me as much as I care about them.	
	AA3. I worry that if I do not work hard to maintain the relationship, the other person will leave me.	
	AA4. I worry that I am not good enough and not worthy of being loved.	
	AA5. When my partner or friends are not around, I feel anxious.	
	AA6. I need a great deal of reassurance to confirm that close others really love me.	

**Table 3:** Conceptual boundaries among the core constructs.

Construct	Focal meaning	Explicit exclusion
Perceived anthropomorphism	Perception that the AI appears or responds in a human-like manner	Not need fulfillment; not dependence
Basic need satisfaction	User’s autonomy, relatedness, and competence experience during interaction	An experiential state, not a property of the AI or a symptom
Problematic emotional dependence	Broad non-clinical composite of dysregulation and emotionally consequential reliance	Not ordinary attachment, diagnosis, or clinical pathology
Loneliness	Perceived insufficiency of offline social connection	Moderator; not assumed to be caused by AI use

### 3.3 Analytical strategy

Data were analyzed in three stages. First, the measurement model was assessed in SmartPLS 4.0 by examining internal consistency reliability, convergent validity, and discriminant validity. Cronbach’s alpha, rho_A, composite reliability, and average variance extracted were evaluated according to conventional PLS-SEM criteria (Hair et al., 2021). Discriminant validity was primarily assessed using the heterotrait–monotrait ratio of correlations (HTMT), because HTMT has been shown to detect discriminant validity problems more sensitively than the traditional Fornell–Larcker criterion (Fornell and Larcker, 1981; Henseler et al., 2015).

Second, the structural paths were estimated using PLS-SEM with bootstrapping based on 5,000 resamples, a commonly used procedure for testing the significance of path coefficients and indirect effects in PLS-SEM (Hair et al., 2021). H1 and H2 were tested through the direct paths from perceived anthropomorphism to basic psychological need satisfaction and from basic psychological need satisfaction to problematic emotional dependence. H3 was tested using the bootstrapped indirect effect of perceived anthropomorphism on problematic emotional dependence through basic psychological need satisfaction (Preacher and Hayes, 2008).

Third, moderation and moderated mediation were tested using PROCESS-style conditional process analysis. The interaction between loneliness and basic psychological need satisfaction was used to test H4. Conditional indirect effects at low, mean, and high levels of loneliness were then estimated to test H5. Finally, attachment anxiety was substituted for loneliness to examine whether the moderation pattern reflected a broader social–emotional vulnerability mechanism (Hayes, 2015).

## 4 Results

### 4.1 Measurement model

The reported composite scores showed high internal consistency and adequate convergent validity. Cronbach’s alpha ranged from 0.913 to 0.950, composite reliability from 0.932 to 0.960, and AVE from 0.697 to 0.801 (Table 4). These values support score reliability and convergence within the specified composites, but they do not establish clinical validity, content validity, or the superiority of a unidimensional PED or NS structure.

**Table 4:** Reliability and convergent validity.

Construct	Cronbach’s α	rho_a	rho_c	AVE
Perceived anthropomorphism (PA)	0.950	0.950	0.960	0.801
Basic psychological need satisfaction (NS)	0.926	0.926	0.942	0.730
Problematic emotional dependence (PED)	0.913	0.914	0.932	0.697
Loneliness (LO)	0.946	0.954	0.957	0.787
Attachment anxiety (AA, robustness)	0.943	0.949	0.955	0.778

Reliability values for PA, NS, PED, and LO are from the main model. AA values are from the alternative moderator model. The same alpha values are reported in the Method section and Results section.

Raw-data indicator diagnostics were consistent with the original reliability estimates. Every item loaded most strongly on its assigned composite; loading ranges were 0.884–0.901 for PA, 0.848–0.863 for NS, 0.820–0.849 for PED, 0.880–0.893 for loneliness, and 0.874–0.895 for attachment anxiety. Item VIFs ranged from 2.21 to 3.78 (Table 5).

**Table 5:** Supplementary raw-data indicator diagnostics.

Construct	Loading range	Item VIF range	Highest loading
PA	0.884–0.901	3.30–3.78	Assigned construct
NS	0.848–0.863	2.53–2.73	Assigned construct
PED	0.820–0.849	2.21–2.53	Assigned construct
LO	0.880–0.893	3.15–3.47	Assigned construct
AA	0.874–0.895	3.00–3.52	Assigned construct

All bootstrapped HTMT estimates were below 0.85 and all upper 95% confidence limits were below 0.70. The largest estimate was LO-AA, 0.619 [0.544, 0.686], followed by PA-NS, 0.606 [0.554, 0.653], and PA-PED, 0.602 [0.549, 0.649] (Table 6).

**Table 6:** HTMT estimates with 2,000-sample bootstrap confidence intervals.

Construct pair	HTMT	95% bootstrap CI
PA-NS	0.606	[0.554, 0.653]
PA-PED	0.602	[0.549, 0.649]
PA-LO	0.199	[0.119, 0.277]
PA-AA	0.142	[0.070, 0.219]
NS-PED	0.579	[0.523, 0.629]
NS-LO	0.361	[0.307, 0.415]
NS-AA	0.420	[0.365, 0.471]
PED-LO	0.209	[0.135, 0.280]
PED-AA	0.205	[0.134, 0.277]
LO-AA	0.619	[0.544, 0.686]

Supplementary maximum-likelihood factor-model checks favored parsimonious single composites. The one-factor PED model fit well, χ^2^(9) = 12.13, *p* = 0.206, CFI = 0.999, RMSEA = 0.023, SRMR = 0.013. A correlated behavioral-dysregulation/relational-reliance model did not improve fit and estimated a factor correlation of 0.9998. The one-factor NS model also fit well; a three-factor alternative produced correlations of 0.976–0.995 among autonomy, relatedness, and competence and had higher information criteria (Table 7).

**Table 7:** Supplementary factor-model comparisons.

Model	*χ*^2^ (df)	CFI	RMSEA	SRMR	AIC	BIC
PED: one factor	12.13 (9)	0.999	0.023	0.013	36.13	90.14
PED: two correlated	12.19 (8)	0.998	0.028	0.013	38.19	96.70
NS: one factor	5.24 (9)	1.000	0.000	0.007	29.24	83.26
NS: three correlated	3.01 (6)	1.000	0.000	0.006	33.01	100.53

### 4.2 Descriptive associations, model fit, and predictive assessment

Pearson correlations among the core variables were consistent with the proposed model. Perceived anthropomorphism was positively correlated with need satisfaction and problematic dependence. Need satisfaction was also positively correlated with problematic dependence and loneliness. Loneliness showed positive but weaker correlations with perceived anthropomorphism and dependency, in Table 8.

**Table 8:** Pearson correlations among core variables.

Construct	PA	NS	PED	LO
PA	1			
NS	0.568***	1		
PED	0.561***	0.532***	1	
LO	0.188***	0.338***	0.194***	1

***p < 0.001. PA = perceived anthropomorphism; NS = basic psychological need satisfaction; PD = problematic emotional dependence; LO = loneliness.

The PLS-SEM model fit indices indicated acceptable model quality for hypothesis testing. The estimated model had an SRMR of 0.090, which is below the commonly used upper bound of 0.10 for PLS-SEM applications, and an NFI of 0.950, exceeding the recommended value of 0.90, in Table 9.

**Table 9:** PLS-SEM model fit indices.

Index	Saturated model	Estimated model
SRMR	0.029	0.090
d_ULS	0.388	3.788
d_G	0.185	0.228
Chi-square	725.647	867.512
NFI	0.958	0.950

Indices are reported from SmartPLS 4.0. PLS-SEM fit indices are interpreted as approximate model quality indicators rather than as the sole basis for model evaluation.

In the standardized composite-score verification, PA explained 32.3% of NS variance (adjusted *R*^2^ = 0.322; *f*^2^ = 0.476). The PED model explained 38.9% of variance (adjusted *R*^2^ = 0.386). Incremental *f*^2^ values were 0.163 for PA, 0.103 for NS, 0.0003 for loneliness, and 0.012 for the interaction. Predictor VIFs were 1.01–1.62. Ten-fold cross-validation yielded positive *Q*^2^_predict values for all six PED indicators (0.232–0.298), and both RMSE and MAE were lower than the training-fold mean benchmark for every indicator (Table 10).

**Table 10:** Ten-fold cross-validated indicator prediction.

Indicator	*Q*^2^_predict	RMSE model	RMSE mean	MAE model	MAE mean
PED1	0.298	1.111	1.326	0.910	1.053
PED2	0.237	1.204	1.378	0.994	1.084
PED3	0.298	1.143	1.365	0.938	1.060
PED4	0.280	1.163	1.370	0.958	1.086
PED5	0.236	1.179	1.350	0.978	1.065
PED6	0.232	1.149	1.312	0.919	1.016

### 4.3 Structural associations

Perceived anthropomorphism was positively associated with basic psychological need satisfaction, *β* = 0.569, *t* = 23.671, *p* < 0.001, 95% CI [0.519, 0.614], consistent with H1. Basic psychological need satisfaction was positively associated with problematic emotional dependence, *β* = 0.317, *t* = 8.477, *p* < 0.001, 95% CI [0.245, 0.391], consistent with H2. PA also retained a direct association with PED, *β* = 0.385, 95% CI [0.312, 0.450]. The direct LO-PED path was not significant, *β* = 0.016, *p* = 0.625 (Table 11). These are concurrent standardized associations.

**Table 11:** Structural path coefficients.

Hyp.	Path	*β*	SE	*t*	*p*	95% CI	Interpretation
H1	PA → NS	0.569	0.024	23.671	<0.001	[0.519, 0.614]	Consistent
H2	NS → PED	0.317	0.037	8.477	<0.001	[0.245, 0.391]	Consistent
-	PA → PED	0.385	0.035	10.866	<0.001	[0.312, 0.450]	–
-	LO → PED	0.016	0.033	0.488	0.625	[−0.045, 0.084]	–

### 4.4 Cross-sectional indirect association

The PA-NS-PED indirect association was 0.180, SE = 0.024, *t* = 7.670, *p* < 0.001, 95% CI [0.137, 0.229]. The direct PA-PED association also remained positive. The estimate is therefore described as a positive concurrent indirect association rather than evidence that need satisfaction temporally mediates an effect of anthropomorphism on dependence (Table 12).

**Table 12:** Structural path coefficients.

Hyp.	Path	Effect	SE	*t*	*p*	95% CI	Interpretation
H3	PA → NS → PED	0.180	0.024	7.670	<0.001	[0.137, 0.229]	Consistent
–	PA → PED (direct)	0.385	–	–	<0.001	[0.312, 0.450]	–
–	PA → PED (total)	0.565	–	–	–	–	–

### 4.5 Moderation and conditional indirect associations

In the full sample, the standardized NS × loneliness interaction was positive, *β* = 0.086, *p* = 0.005, 95% CI [0.026, 0.146]. The standardized NS-PED slope increased from approximately 0.232 at low loneliness to 0.405 at high loneliness. The conditional indirect estimates were 0.132, 0.181, and 0.230 at low, mean, and high loneliness, respectively, and the index was 0.049, 95% bootstrap CI [0.016, 0.084]. Thus H4 and H5 were consistent with the full-sample data. After the platform-label correction, the pooled estimate for the five named companion/character platforms was smaller and nonsignificant, *N* = 583, *β* = 0.057, *p* = 0.083, 95% CI [−0.007, 0.121], and its conditional-indirect index included zero (Tables 13, 14).

**Table 13:** Platform sensitivity of the NS × loneliness association.

Analysis	*N*	Interaction *β*	*p*	95% CI
Full sample	666	0.086	0.005	[0.026, 0.146]
Five named companion/character platforms	583	0.057	0.083	[−0.007, 0.121]
Maoxiang	114	0.173	0.012	[0.039, 0.307]
Xingye	144	−0.048	0.464	[−0.178, 0.082]
Zhumengdao	134	0.102	0.147	[−0.036, 0.240]
Replika	96	0.104	0.228	[−0.066, 0.274]
Character.AI	95	−0.085	0.353	[−0.265, 0.096]
Other platform category	83	0.373	<0.001	[0.192, 0.553]
Companion-purpose records	631	0.087	0.006	[0.025, 0.149]
Named-platform vs. other difference	666	−0.308	0.001	[−0.492, −0.124]

The last row is the NS × loneliness × named-platform coefficient from a pooled observed-group regression; a negative value indicates weaker moderation across the five named platforms than in the heterogeneous ‘other’ category. Individual-platform estimates are exploratory and imprecise because subgroup sizes are modest. Table coefficients use a rescaled standardized-product term; figure annotations show the unrescaled coefficient for the product of standardized NS and loneliness.

**Table 14:** Conditional indirect associations.

Sample/level	Estimate	95% bootstrap CI	Interpretation
Full: low loneliness	0.132	[0.080, 0.185]	CI excludes zero
Full: mean loneliness	0.181	[0.137, 0.228]	CI excludes zero
Full: high loneliness	0.230	[0.171, 0.294]	CI excludes zero
Full: index	0.049	[0.016, 0.084]	CI excludes zero
Five named platforms: index	0.032	[−0.002, 0.069]	CI includes zero
Maoxiang: index	0.101	[0.049, 0.163]	CI excludes zero
Other category: index	0.242	[0.121, 0.369]	CI excludes zero

### 4.6 Exploratory robustness check using attachment anxiety

Attachment anxiety was selected as an alternative moderator because it represents concern about rejection and relational availability, whereas loneliness represents perceived insufficiency of social connection. The standardized AA × NS interaction was positive, *β* = 0.084, *p* = 0.006, 95% CI [0.024, 0.144], and the index of moderated indirect association was 0.048, 95% bootstrap CI [0.015, 0.084]. These results indicate that higher attachment anxiety strengthens the indirect association via basic psychological need satisfaction. In other words, individuals with higher attachment anxiety are more likely to translate the psychological need satisfaction derived from AI companions into problematic emotional dependence. Furthermore, this exploratory analysis provides supplementary evidence supporting the robustness of the primary model (Table 15).

**Table 15:** Exploratory attachment-anxiety robustness analysis.

Effect	Estimate	SE	*t*	*p*	95% CI
AA × NS → PED	0.084	0.030	2.754	0.006	[0.024, 0.144]
Indirect association at low AA	0.128	–	–	–	[0.071, 0.180]
Indirect association at mean AA	0.175	–	–	–	[0.130, 0.222]
Indirect association at high AA	0.223	–	–	–	[0.166, 0.283]
Index	0.048	–	–	–	[0.015, 0.084]

## 5 Discussion

### 5.1 Overview of findings

This study examined how perceived anthropomorphism is associated with problematic emotional dependence among AI companion users and whether basic psychological need satisfaction and loneliness help explain when this association becomes strongest. Using data from 666 AI companion users in China, the study supported all five hypotheses. Perceived anthropomorphism, interaction-specific basic psychological need satisfaction, and problematic emotional dependence were positively associated in this sample, and the principal paths replicated across PLS and composite-score estimators. The full-sample concurrent indirect association was stronger at higher loneliness, and an exploratory attachment-anxiety model showed a parallel pattern. Crucially, the loneliness interaction was absent in the dedicated/character-platform subgroup and significantly weaker than in the remaining platforms. The positive main associations are therefore more robust than the moderation claim. None of these findings identifies temporal direction, causal mediation, clinical pathology, or actual replacement of human relationships (T’ng et al., 2022).

Anthropomorphism should not be interpreted as inherently harmful. Its perceived humanness was positively associated with both need satisfaction and dependence, but the observed moderation pattern varied across the platform ecology. The same anthropomorphic affordances may support benign engagement, perceived support, or dysregulated reliance depending on user circumstances and interaction patterns. The study therefore identifies associations worthy of further testing, not a universal design effect or a general loneliness-amplification mechanism.

The central conclusion is therefore consistent across the abstract, results, and discussion. Perceived anthropomorphism does not merely make AI companions attractive or engaging; it makes them need-satisfying, personally responsive, and relationally meaningful. This need satisfaction partly explains why users may become problematic dependence. This interpretation is consistent with prior work showing that anthropomorphism can shape human–AI attachment and social consequences, and that AI companions can provide users with emotional validation, perceived understanding, and a sense of being heard (De Freitas et al., 2024; Guingrich and Graziano, 2025; Hermann, 2022). Loneliness intensifies this process because AI-provided need satisfaction may carry greater substitutive value for users whose real-world social needs are insufficiently met. This is consistent with evidence that loneliness and social vulnerability shape the psychological outcomes of AI companion use (Ciriello et al., 2026; Lai et al., 2025). The robustness analysis with attachment anxiety further indicates that the moderation pattern is not limited to loneliness alone, but may reflect a broader vulnerability among users with heightened relational insecurity (Ciriello et al., 2026; Knox et al., 2025; Liu et al., 2026).

### 5.2 Theoretical contributions

First, the study places companion-oriented conversational AI within SDT while preserving SDT’s distinction among autonomy, competence, and relatedness. The broad NS composite provides an interaction-level estimate, but the paper no longer claims that all three needs contribute equally. The supplementary dimension model yielded small relatedness and competence coefficients and a nonsignificant autonomy coefficient under very high inter-dimension correlations; stronger multi-item measures are needed before unique dimensional contributions can be inferred (Parent, 2023; Przybylski et al., 2010).

Second, the study reframes the previously claimed need-substitution mechanism as a testable future explanation. The present data show that perceived anthropomorphism, need satisfaction, and problematic dependence covary. They do not show that AI-based need satisfaction displaced offline fulfillment or reduced human interaction. A direct test would require measures of offline need satisfaction, relative reliance on AI versus people, and change over time (De Freitas et al., 2024; Liu et al., 2026).

Third, loneliness is positioned as a possible moderator of a concurrent association rather than as a deterministic risk factor. Its full-sample interaction, non-replication in dedicated/character platforms, and exploratory attachment-anxiety result collectively show why relational vulnerability cannot be inferred from one pooled coefficient. Future work should preregister platform-stratified tests and directly measure platform affordances and user motives (Yao et al., 2025).

### 5.3 Practical implications for developers and policymakers

Developers should evaluate anthropomorphic features in relation to user control and interaction context rather than treating human-like design as intrinsically unsafe. Relevant safeguards include clear disclosure that the system is artificial, controls for memory and personalization, easy deletion and export of user data, frictionless disengagement, non-manipulative notifications, usage dashboards, and prompts that support reflection or offline contact when users report distress. Systems should avoid exclusivity cues, guilt-inducing messages, or engagement strategies that imply the AI needs the user (Hudon and Stip, 2025; Kaffee et al., 2025; Knox et al., 2025).

Policymakers and platform governance teams can require age-appropriate design, transparent advertising of relational capabilities, auditable risk assessments for emotional-reliance features, accessible complaint mechanisms, and independent evaluation of high-risk engagement patterns. Safeguards should be proportionate and should not deny companionship to lonely users. The aim is to preserve supportive use while reducing manipulative or dependency-promoting design incentives (Baggot, 2025; Boine, 2023; De Freitas et al., 2024).

Educators, counselors, and mental-health practitioners should interpret self-reported AI reliance within the user’s broader social ecology. The revised PED measure is not a diagnostic tool (Seeger et al., 2021). It may help identify experiences that warrant conversation, but clinical screening or intervention requires validated instruments, functional-impairment evidence, and professional judgment (Ciriello et al., 2026; Lai et al., 2025; Shen et al., 2026).

### 5.4 Limitations and future research

Several limitations should be acknowledged. First, the cross-sectional design cannot establish temporal order or causal mediation. Reverse and reciprocal associations are plausible; longitudinal panel and experimental designs are needed. Second, all focal variables were self-reported in one survey. Shared method, evaluative tone, and semantic overlap may inflate associations even when HTMT is below conventional cutoffs. Third, the Chinese context and platform ecology limit generalizability. Cross-cultural replication should measure, rather than presume, cultural mechanisms.

## 6 Conclusion

Among adults in China reporting recent use of listed AI-companion or conversational-AI products, perceived anthropomorphism was positively associated with interaction-specific basic psychological need satisfaction and with a broad, non-clinical measure of problematic emotional dependence. A loneliness-contingent indirect association appeared in the full sample but not among dedicated/character-platform users. The robust contribution is therefore the positive cross-sectional association among the core constructs; the moderation finding is platform-contingent and requires replication. Responsible interpretation and design require attention to construct validation, platform ecology, recruitment provenance, and longitudinal evidence.

## Data Availability

The raw data supporting the conclusions of this article will be made available by the authors, without undue reservation.
